# Antitumor Effects of Pegylated Zinc Protoporphyrin-Mediated Sonodynamic Therapy in Ovarian Cancer

**DOI:** 10.3390/pharmaceutics15092275

**Published:** 2023-09-04

**Authors:** Jia Li, Zheng Hu, Jiwei Zhu, Xin Lin, Xu Gao, Guixiang Lv

**Affiliations:** 1 Department of Biochemistry and Molecular Biology, Harbin Medical University, Basic Medical Institute of Heilongjiang Medical Sciences Academy, Harbin 150086, China; lijia2829@163.com (J.L.); linxin990831@126.com (X.L.); gaoxu6712@163.com (X.G.); 2 School of Medicine and Health, Harbin Institute of Technology, Harbin 150080, China; huzheng@hit.edu.cn; 3 Department of Forensic Medicine, Harbin Medical University, Harbin 150086, China; zhujw@ems.hrbmu.edu.cn

**Keywords:** pegylated zinc protoporphyrin, sonodynamic therapy, reactive oxygen species, apoptosis, antitumor effects

## Abstract

Sonodynamic therapy (SDT) induces reactive oxygen species (ROS) to kill tumor cells. Heme oxygenase-1 (HO-1), as an important antioxidant enzyme, resists killing by scavenging ROS. Zinc protoporphyrin (ZnPP) not only effectively inhibits HO-1 activity, but also becomes a potential sonosensitizer. However, its poor water solubility limits its applications. Herein, we developed an improved water-soluble method. It was proved that pegylated zinc protoporphyrin-mediated SDT (PEG-ZnPP-SDT) could significantly enhance ROS production by destroying the HO-1 antioxidant system in ovarian cancer. Increased ROS could cause mitochondrial membrane potential collapse, release cytochrome c from mitochondria to the cytoplasm, and trigger the mitochondrial–caspase apoptotic pathway. In conclusion, our results demonstrated that PEG-ZnPP-SDT, as a novel sonosensitizer, could improve the antitumor effects by destroying the HO-1 antioxidant system. It provided a new therapeutic strategy for SDT to treat cancers, especially those with higher HO-1 expression.

## 1. Introduction

Ovarian cancer is one of the most common malignant tumors in the female reproductive system, of which the global incidence is second only to cervical and endometrial cancers, and the mortality rate is ranked second among other female malignant tumors [1]. Although traditional modalities such as surgery, chemotherapy, and radiotherapy are constantly improving, the treatment outcomes are not satisfactory. Therefore, there is an urgent need to explore effective clinical treatment methods.

Sonodynamic therapy (SDT) is a promising physical therapy developed on the basis of photodynamic therapy (PDT) [2]. It uses low-intensity ultrasound to stimulate sonosensitizers accumulated in tumor cells and destroy tumor cells by generating reactive oxygen species (ROS) [3]. Currently, SDT has shown great potential in cancer therapy due to its good therapeutic effects, which have been applied to liver cancer [4], breast cancer [5], melanoma [6], lung cancer [7], oral cancer [8], pancreatic cancer [9], glioma [10], osteosarcoma [11], and other malignant tumors.

Ultrasonic cavitation is the main trigger of SDT-induced ROS. When traveling through liquid/tissues, ultrasound forces the liquid to oscillate in the acoustic field. With the increase in acoustic pressure, this oscillation becomes unstable and eventually leads to the burst of cavitation bubbles. At the time of implosion, extreme temperatures and pressures are generated. The sonosensitizers absorb and transfer energy either to the ground-state oxygen, leading to the production of ^1^O_2_ (Type II reaction), or to the adjacent biological substrates, leading to the production of other ROS, such as ·O_2_^−^, ·OH, H_2_O_2_ by transferring protons, or electrons (Type I reaction) [12].

There are three essential elements that affect SDT outcomes: ultrasound, sonosensitizer, and molecular oxygen, in which sonosensitizer plays the most critical role. At present, sonosensitizers are mainly porphyrins and their derivatives, among which metalloporphyrin-mediated SDT plays a vital role in the treatment of malignant tumors [13,14]. MnTTP, encapsulated with human serum albumin (HSA), exhibited strong ROS-activated behavior. Moreover, it was used as a novel nanosonosensitizer for deep-tissue imaging-guided SDT [15]. Xu et al. constructed a manganese porphyrin-based metal–organic framework (Mn-MOF), and found that it could not only catalyze the production of O_2_ from H_2_O_2_ to alleviate tumor hypoxia, but also reduce the expression of GSH and GPX4, leading to better antitumor effects in H22 and 4T1 tumor-bearing mice [16]. 

This article aims to reveal the mechanisms of pegylated zinc protoporphyrin-mediated SDT (PEG-ZnPP-SDT) in the treatment of ovarian cancer and provide new ideas for the treatment of ovarian cancer.

## 2. Materials and Methods

### 2.1. Chemicals and Antibodies

Protoporphyrin IX (PpIX, P8293), zinc protoporphyria (ZnPP, 282820), tetrahydrofuran (THF, 401757), triethylamine (TEA, T0886), N-acetylcysteine (NAC, A7250), nicotinamide adenine dinucleotide phosphate (NADPH, 621706), hemin (51280), and 2-(4-Amidinophenyl)-6-indolecarbamidine dihydrochloride (DAPI, D8417) were purchased from Sigma (Shanghai, China). Isobutyl chloroformate (01086117) was purchased from Adamas (Shanghai, China). Ethylene diamine (2143) and chloroform (3084) were purchased from Damao (Tianjin, China). The methoxy (polyethylene glycol) succinimidyl carboxyl methyl ester (mPEG-NHS, PS1-H-1K), with an average molecular weight of 1000, was purchased from Ponsure (Shanghai, China). Zinc acetate (Z111245) was purchased from Aladdin (Shanghai, China). Cell Counting Kit-8 (CCK-8,C0039), 2′,7′-dichlorofluorescein diacetate (DCFH-DA, S0033S), and Mito-Tracker Green (C1048) were purchased from Beyotime (Shanghai, China). Anti-PCNA (60097) was purchased from Proteintech (Wuhan, China). Anti-cytochrome c (ab133504) was purchased from Abcam (Shanghai, China), and anti-cleaved caspase-3 (9661) was purchased from Cell Signaling Technology (Shanghai, China).

### 2.2. Synthesis of PEG-ZnPP

The 10 mg of PpIX was dissolved in 5 mL of THF, and 250 μL of TEA was added with stirring using an ice bath. After that, isobutyl chloroformate (230 μL) was added by dropwise under stirring, and the reaction was continued at 0 °C for 2 h. Then, the reaction mixture was centrifuged at 5000 rpm for 5 min and the supernatant was collected. The 120 μL of ethylene diamine was added with stirring for 24 h. The reaction mixture was dried at 70 °C for 4 h, washed 3 times with pre-cooled distilled water, and centrifuged at 5000 rpm for 5 min. Then, the supernatant was discarded completely. The 5 mL of chloroform and 18.6 mg of mPEG-NHS were added with stirring for 2 h. Then, 30 mg of zinc acetate was added with stirring for 24 h. The solution was dried at 70 °C for 3 h, re-suspended with 2 mL of distilled water, and centrifuged at 5000 rpm for 5 min. The supernatant was collected, purified, and identified by size exclusion chromatography (AKTA Avant 150; GE Healthcare, Uppsala, Sweden) using Sephadex LH-60 column (Solarbio S4580, Beijing, China) and high-performance liquid chromatography (HPLC; Agilent 1200, Santa Clara, CA, USA) reported by Sahoo et al. [17]. The fraction was lyophilized to obtain pegylated zinc protoporphyrin (PEG-ZnPP) using a VirTis Advantage Lyophilizer (SP Scientific, Gardiner, NY, USA).

### 2.3. Spectral Characteristics and Water Solubility Analysis of PEG-ZnPP

PEG-ZnPP was dissolved in serum-free DMEM. The UV–visible absorption spectra of PEG-ZnPP in the concentration range of 0.5–2.0 μM were measured using an Ocean Optics QE65000 spectrometer (Dunedin, FL, USA). The photoluminescence spectra in the concentration range of 0.5–2.0 μM were measured using an Ocean Optics USB 4000 spectrometer (Dunedin, FL, USA). The water solubility of the synthesized PEG-ZnPP (1 mM) was compared with ZnPP (1 mM) in the same treatment at 4 °C. The HO-1 activity assay was consistent with the approach reported by Sahoo et al. [17]. In brief, 1.0 mg of HO-1 protein derived from mouse spleen, 3.0 mg of biliverdin reductase derived from mouse liver, 333 μM NADPH, 33 μM hemin, and 1 μM PEG-ZnPP or ZnPP were mixed in 1.0 mL of 90 mM potassium phosphate buffer (pH 7.4). After being incubated at 37 °C for 15 min, the reaction was terminated by adding 33 μL of HCl. Then, the bilirubin formation was extracted with 1 mL of chloroform, measured using an Ocean Optics QE65000 spectrometer, and calculated by the difference of absorbance at 465 nm and 530 nm with a molar extinction coefficient of 40 mM^−1^ cm^−1^.

### 2.4. Cell Culture

Human ovarian cancer cell line SKOV3 was provided by the Cell Bank of the Type Culture Collection of Chinese Academy of Sciences (Shanghai, China). The cells were cultured in Dulbecco’s modified Eagle’s medium (high sugar, DMEM) containing fetal bovine serum (10%), penicillin (100 μg/mL), and streptomycin (100 μg/mL) in a humidified incubator at 37 °C, 95% air, and 5% CO_2_.

### 2.5. Cytotoxicity Assay of PEG-ZnPP

SKOV3 cells were seeded at a density of 1 × 10^4^ cells per well in a 96-well plate, and PEG-ZnPP with different concentrations of 0, 10, 20, 40, and 80 μM were added to 96-well plate and incubated for 24 h. The cell viability was detected by CCK-8 assay. The optical density (OD) values of each well were measured at 450 nm using a TECAN Infinite 200 PRO microplate reader (Männedorf, Switzerland).

### 2.6. Cellular Uptake of PEG-ZnPP

SKOV3 cells were seeded at a density of 1 × 10^4^ cells per well for a 96-well plate and 5 × 10^4^ cells per well for a 24-well plate. The cells were divided into six groups, including 0, 1, 3, 6, 9, and 12 h. The fluorescence intensity of PEG-ZnPP in each group of cells in the 96-well plate was, respectively, measured using a TECAN Infinite 200 PRO microplate reader or a BD Accuri C6 Plus flow cytometer (Franklin Lakes, NJ, USA).

### 2.7. Ultrasonic System

The ultrasonic system was similar to that described previously [18]. In brief, for in vitro experiments, tumor cells were placed in a cell culture dish on an ultrasonic transducer. For in vivo experiments, an ultrasonic transducer with a front surface was used to treat tumors with an acoustic coupling gel. The ultrasound frequency of 1.1 MHz, duty cycle of 10%, ultrasound intensity of 1 W/cm^2^ and ultrasound time of 120 s were used for treatments.

### 2.8. Intracellular Sublocalization of PEG-ZnPP

SKOV3 cells were seeded in a cell culture dish at a density of 2 × 10^5^ cells. The 20 μM PEG-ZnPP was added and incubated for 9 h. The cells were washed twice with PBS and incubated with a 50 nM Mito-Tracker Green for 30 min. The nuclei were stained with 1 µg/mL of DAPI for 5 min, and finally, fluorescence imaging was performed using a BioTek Live Cell Imager (Winooski, VT, USA).

### 2.9. SDT Treatment

SKOV3 cells were seeded at a density of 1 × 10^4^ cells per well in a 96-well plate and incubated overnight. The cells were divided into eight groups with Control, US alone, PpIX alone, ZnPP alone, PEG-ZnPP alone, US + PpIX (ultrasound combined with PpIX), US + ZnPP (ultrasound combined with ZnPP), and US + PEG-ZnPP (ultrasound combined with PEG-ZnPP). The concentration of sonosensitizers was 20 μM, and the incubation time was 9 h. The cell survival of SKOV3 cells was measured by CCK-8 assay after ultrasound treatment. The OD values of each well were measured at 450 nm using a TECAN Infinite 200 PRO microplate reader.

### 2.10. Intracellular ROS Measurement

To measure intracellular ROS, cells were pretreated with 5 mM NAC for 1 h prior to ultrasound treatment. Then, 10 μM DCFH-DA was added and incubated at 37 °C for 10 min. After that, cells were sonicated, incubated for 20 min, and washed 3 times with PBS. Fluorescence imaging was performed using a BioTek Live Cell Imager. The ROS production was calculated using the IPP 5.0 software (Media Cybernetics, Bethesda, MD, USA).

### 2.11. Mitochondrial Membrane Potential Detection

The detection was carried out using JC-1 (Beyotime C2003S, Shanghai, China). Briefly, immediately after ultrasound treatment, the cells were incubated with JC-1 probe for 20 min, and fluorescence imaging was performed using a BioTek live cell imager. Fluorescence intensity was calculated using the IPP software.

### 2.12. Immunofluorescence

Immunofluorescence experiments were similar to that in our previous study with minor modifications [18]. Five hours after sonication, cells were incubated with 200 nM Mito-Tracker Green for 30 min, blocked with 1% BSA for 20 min, and incubated with anti-cytochrome c (1:100) at 4 °C overnight. Cells were incubated with Alexa Fluor 555 secondary antibody (1:500; Beyotime P0179, Shanghai, China) for 1 h. Then, DAPI was added and incubated for 15 min. Fluorescence images were taken at 555 nm, 490 nm, and 364 nm, respectively, using a BioTek Live Cell Imager.

### 2.13. Tumor Treatment Model

BALB/c nude mice (6–8 weeks old, approximately 20 g per mouse) were purchased from SLAC (Changsha, Hunan, China). Then, 100 μL DMEM containing SKOV3 cells (3 × 10^6^ cells) was inoculated subcutaneously on the right lower side of the mice. Seven days after inoculation, the mice were randomly divided into eight groups, with six mice in each group, for SDT treatment. PpIX, ZnPP, and ZnPP-PEG were injected into the tail vein at the same molality concentration of 9 μmol/kg per mouse. Mice were sacrificed after 15 days of SDT treatment. Body weight of mice and tumor volume were measured daily. The tumor volume (*V*) was calculated using the formula *V* = π/6 × *L* × *S*^2^ (*L* and *S* were, respectively, long and short diameters of tumors).

### 2.14. Immunohistochemistry

As described in our previous study [18], tissue sections were retrieved in 10 mM EDTA buffer (pH 6.0) for 20 min after dewaxing and rehydration and immersed in 3% hydrogen peroxide for 15 min. Then, the sections were blocked with 5% BSA for 1 h, stained with anti-PCNA and anti-cleaved caspase-3 (both of dilution ratio 1:100), and incubated overnight at 4 °C. After that, the sections were incubated with secondary antibody for 30 min and observed under a light microscope. Immunopositive cells were calculated using the IPP software.

### 2.15. Statistical Analysis

Statistical analysis was performed using the Origin 8.0 software (Berkeley, CA, USA). Data are presented as mean ±SD (standard deviation) from at least three independent experiments. Differences between groups were analyzed using one-way analysis of variance. * *p* < 0.05, ** *p* < 0.01, and ^&^ *p* < 0.05 were considered statistically significant.

## 3. Results

### 3.1. Identification of PEG-ZnPP

**Figure 1:**
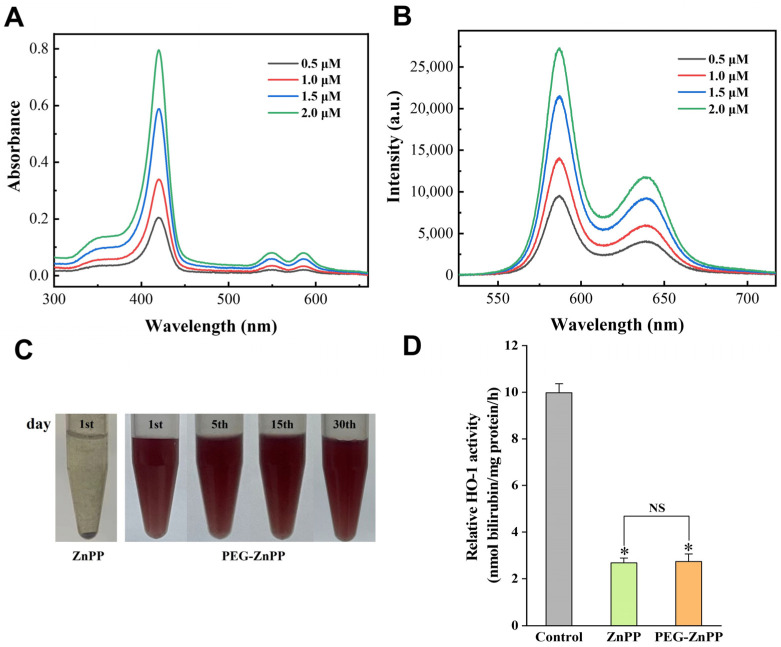
Spectral characteristics and water solubility analysis of PEG-ZnPP. (**A**) The absorption spectra of PEG-ZnPP in the concentration range of 0.5–2.0 μM exhibited three bands at 420 nm, 548 nm, and 584 nm, with the maximum absorption peak at 420 nm. (**B**) The fluorescence spectra of PEG-ZnPP in the concentration range of 0.5–2.0 μM exhibited two bands at 586 nm and 637 nm, with the maximum emission peak at 586 nm. (**C**) PEG-ZnPP (1 mM) exhibited better water solubility compared with ZnPP (1 mM) at 4 °C during a period of 30 days. (**D**) PEG-ZnPP had better inhibitory effects on the activity of HO-1 compared with ZnPP. * *p* < 0.05 vs. control group, and NS with no significant difference between two groups. Data are expressed as mean ± standard deviation of three independent experiments.

Sonosensitizers play an important role in the treatment of SDT, but there is also a problem of poor water solubility, especially for metalloporphyrins (ZnPP, etc.). Hererin, we performed the modification of ZnPP by conjugating water-soluble polyethylene glycol (PEG). In order to improve efficiency and success, we conjugated PpIX with PEG prior to the chelation of zinc ions (Zn^2+^) and eventually synthesized PEG-ZnPP (see Materials and Methods for details). The spectral analysis showed that PEG-ZnPP exhibited three bands at 420 nm, 548 nm, and 584 nm, with the maximum absorption peak at 420 nm, which was consistent with the spectral characteristics of ZnPP (Figure 1A). Furthermore, two fluorescence emission peaks were observed at 586 nm and 637 nm, with the maximum emission peak at 586 nm (Figure 1B). Then, the water solubility of PEG-ZnPP was tested. The high concentration (1 mM) of PEG-ZnPP had better water solubility, and the aqueous solution remained unchanged for 30 days, while ZnPP was completely insoluble in water, indicating excellent stability of PEG-ZnPP (Figure 1C). Finally, we examined the antioxidant capacity of PEG-ZnPP and found that, compared with the control group, the inhibition of HO-1 activity by PEG-ZnPP and ZnPP groups was 73.2% and 72.6%, respectively (Figure 1D). The above results indicated that PEG-ZnPP had better water solubility as well as significant inhibitory effects on HO-1 activity.

### 3.2. Determination of the Optimal SDT Parameters for PEG-ZnPP

**Figure 2:**
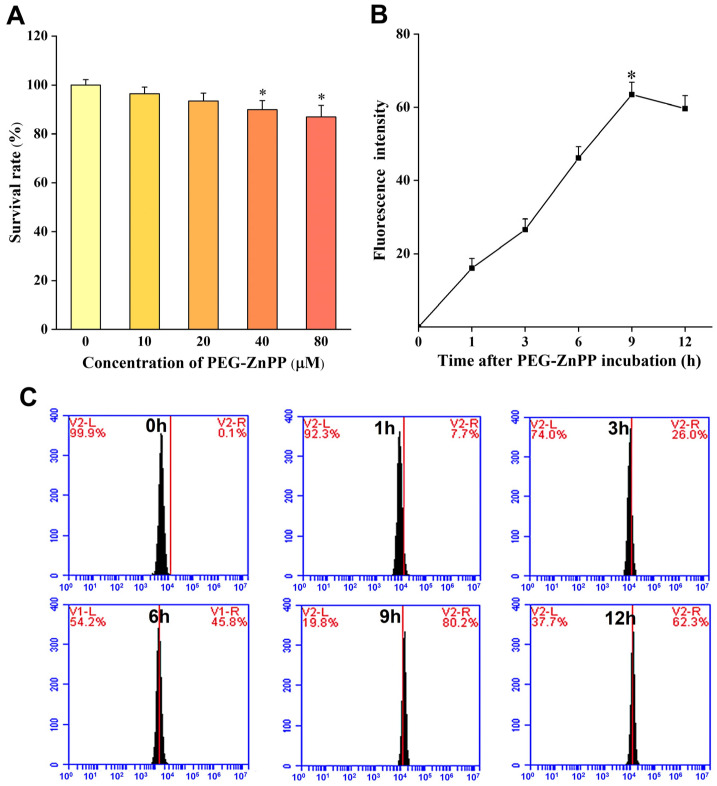
Determination of the optimal SDT parameters for PEG-ZnPP in SKOV3 cells. (**A**) Cytotoxic effects of different concentrations of PEG-ZnPP (0–80 μM) on SKOV3 cells for 24 h. PEG-ZnPP in the range of 0–20 μM had no significant effects on cell viability. Fluorescence intensities of PEG-ZnPP at different incubation time were detected by fluorescent microplate reader (**B**) and flow cytometry (**C**) in SKOV3 cells. The fluorescence intensities of PEG-ZnPP increased continuously and reached a peak at the 9th hour, and then began to decrease gradually. * *p* < 0.05 vs. control group and data are the mean ± standard deviation of three independent experiments.

In order to achieve better therapeutic outcomes using SDT in ovarian cancer, the optimal SDT parameters for PEG-ZnPP should be determined. Firstly, we carried out the cytotoxicity effects assay of PEG-ZnPP. The cells were treated with different concentrations of PEG-ZnPP (0, 10, 20, 40, and 80 μM) for 24 h and then were measured by the CCK-8 assay. The results showed that PEG-ZnPP in the range of 0–20 μM had no significant effects on cell viability, with a cell survival rate of 93.46 ± 3.24% at 20 μM (Figure 2A). However, its cytotoxicity effects on SKOV3 cells increased when the concentration of PEG-ZnPP was above 40 μM (*p* < 0.05). Therefore, 20 μM was considered the optimal concentration for PEG-ZnPP-mediated SDT (PEG-ZnPP-SDT). Subsequently, we detected the cellular uptake of PEG-ZnPP by SKOV3 cells. The fluorescence intensities were, respectively, detected by a fluorescent microplate reader (Figure 2B) and flow cytometry (Figure 2C). The results showed that the fluorescence intensity of PEG-ZnPP increased continuously, reached a peak at the ninth hour, and then began to decrease gradually. Therefore, the ninth hour was considered the optimal incubation time for PEG-ZnPP-SDT.

### 3.3. The Subcellular Localization and SDT Efficacy

**Figure 3:**
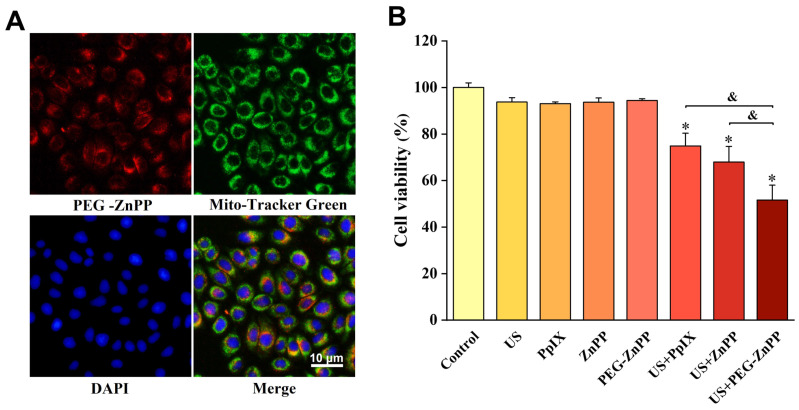
Subcellular localization of PEG-ZnPP and SDT efficacy in SKOV3 cells. (**A**) Representative fluorescence images of PEG-ZnPP in SKOV3 cells. PEG-ZnPP was mainly localized on mitochondria. (**B**) Comparison of different sonosensitizer-mediated SDT on the viability of SKOV3 cells. The US + PEG-ZnPP group significantly inhibited cell viability compared with other groups. * *p* < 0.05 vs. control group and ^&^ *p* < 0.05 between two groups. Data are the mean ± standard deviation of three independent experiments.

Our previous studies found that mitochondria were important sites for the accumulations of sonosensitizers [18]. Therefore, we first determined the localization of PEG-ZnPP in SKOV3 cells. The results are shown in Figure 3A, where PEG-ZnPP was mainly localized on mitochondria, as expected. We then carried out SDT treatment using the optimal concentration and incubation time of PEG-ZnPP as described above. At the same time, we also compared the efficacy of US + PEG-ZnPP, US + ZnPP, and US + PpIX. The results suggested that US + PEG-ZnPP, US + ZnPP, and US + PpIX significantly inhibited the viability of SKOV3 cells, while the other groups had no effects on the viability of SKOV3 cells, and the decrease in cell viability in the US + PEG-ZnPP group (51.6 ± 6.4%) was more significant than that of the US + ZnPP and US + PpIX groups (67.9 ± 6.7% and 74.8 ± 5.4%) (Figure 3B). These results indicated that the efficacy of PEG-ZnPP was significantly better than that of ZnPP and PpIX in SDT.

### 3.4. PEG-ZnPP-Mediated ROS Generation

**Figure 4:**
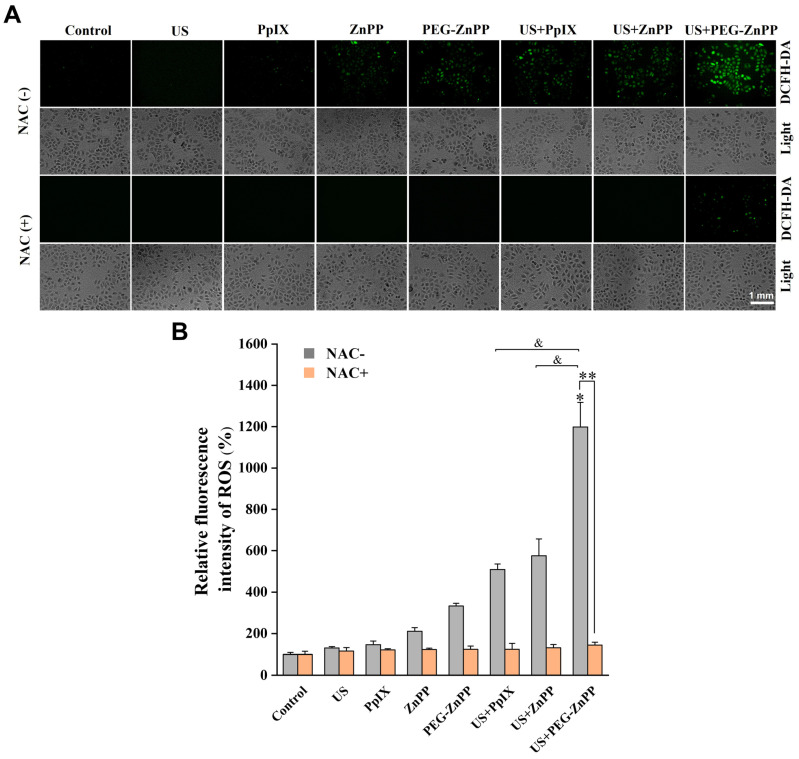
PEG-ZnPP-SDT induces ROS generation. (**A**) Representative fluorescence images of ROS in SKOV3 cells after SDT treatment. (**B**) The relative levels of intracellular ROS were detected by the fluorescent intensities quantified with optical density (OD) values. The ROS production in the US + PEG-ZnPP group increased more significantly than that in other groups, which could be suppressed in the period of NAC (ROS inhibitor). * *p* < 0.05 vs. control group and ^&^ *p* < 0.05 between two groups. ** *p* < 0.01 NAC-untreated vs. NAC-treated US + PEG-ZnPP groups. Data are the mean ± standard deviation of three independent experiments.

Our previous studies showed that SDT-induced cell death was closely related to ROS [4,6]. Therefore, we detected intracellular ROS levels after SDT treatment by staining with DCFH-DA. As shown in Figure 4, the increase in ROS was observed in either US + PpIX compared with PpIX, US + ZnPP compared with ZnPP, or US + PEG-ZnPP compared with PEG-ZnPP groups. In addition, the ROS production in the US + PEG-ZnPP group (1198.9 ± 119%) increased more significantly than that in both the US + ZnPP (575.6 ± 81.2%) and US + PpIX (509.04 ± 26.3%) groups. However, the generation of ROS could be significantly suppressed when NAC (ROS inhibitor) was added. These results indicated that PEG-ZnPP could promote the production of ROS more effectively than that of ZnPP and PpIX in SDT.

### 3.5. PEG-ZnPP-SDT Triggered the Collapse of Mitochondrial Membrane Potential via ROS

**Figure 5:**
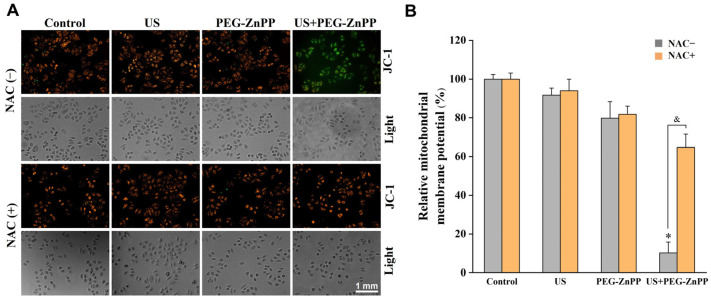
PEG-ZnPP-SDT triggered the collapse of mitochondrial membrane potential via ROS. (**A**) Representative fluorescence images of mitochondrial membrane potential changes (Δψm) in SKOV3 cells after SDT treatment. (**B**) Relative fluorescence intensities of Δψm were quantified by OD values. The relative Δψm in the US + PEG-ZnPP group decreased more significantly than that in other groups, which could be suppressed in the period of NAC (ROS inhibitor). * *p* < 0.05 vs. control group and ^&^ *p* < 0.05 between two groups. Data are the mean ± standard deviation of three independent experiments.

To explore the relationship between PEG-ZnPP-SDT and mitochondrial damage, the changes in the mitochondrial membrane potential (Δψm) in SKOV3 cells were detected by the JC-1 assay. As shown in Figure 5, the relative Δψm of the US alone or the PEG-ZnPP alone groups decreased by 10–20% compared with the control group, respectively. However, the relative Δψm of the US + PEG-ZnPP group decreased by 89.8 ± 5.5%. After the cells were treated with NAC, the relative Δψ of the US + PEG-ZnPP group was significantly increased compared with the NAC-untreated one. These results indicated that PEG-ZnPP-SDT was the main cause of mitochondrial membrane potential collapse via ROS.

### 3.6. PEG-ZnPP-SDT Activated Mitochondria–Caspase Pathway via ROS

**Figure 6:**
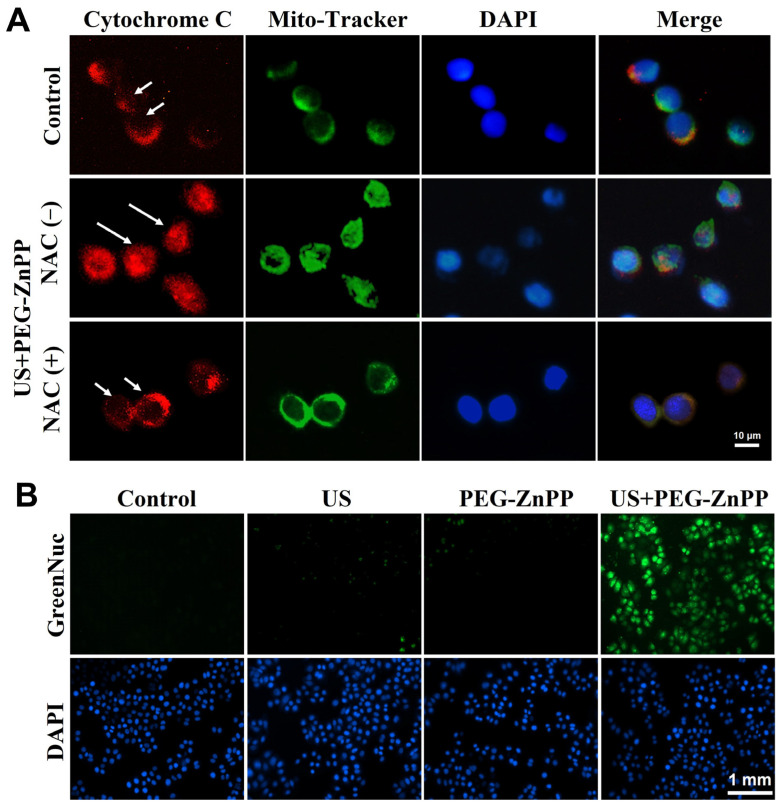
PEG-ZnPP-SDT activated mitochondria–caspase pathway via ROS. (**A**) Representative fluorescence images of cytochrome c translocation in SKOV3 cells after SDT treatment. In the NAC-untreated US + PEG-ZnPP group, cytochrome c (red fluorescence) diffused in the cytoplasm (long arrow), while it was immobilized in the mitochondria (short arrow) in the control and NAC-treated US + PEG-ZnPP groups. (**B**) Expression of caspase-3 in SKOV3 cells was measured with the GreenNuc kit. The activity of caspase-3 (green fluorescence) in the US + PEG-ZnPP group was significantly higher than that in other groups.

To further investigate how ROS participated in PEG-ZnPP-mediated apoptosis, the effects of ROS on the mitochondria–caspase pathway in SKOV3 cells were detected. As shown in Figure 6A, red fluorescence was observed in the cytoplasm (long arrow) in the NAC-untreated US + PEG-ZnPP group, and no significant changes were observed in the control and NAC-treated US + PEG-ZnPP groups (short arrow), indicating that ROS were involved in the release of cytochrome c from mitochondria to the cytoplasm. The activity of caspase-3 (a key apoptosis executor) was also detected by GreenNuc kit. As shown in Figure 6B, the activity of caspase-3 in the US + PEG-ZnPP group was significantly higher than that in the control, US, and PEG-ZnPP groups. These data suggested that PEG-ZnPP-SDT indeed induced mitochondrial–caspase-dependent apoptosis via ROS.

### 3.7. PEG-ZnPP-SDT Exhibited Significant Antitumor Effects in Ovarian Cancer

**Figure 7:**
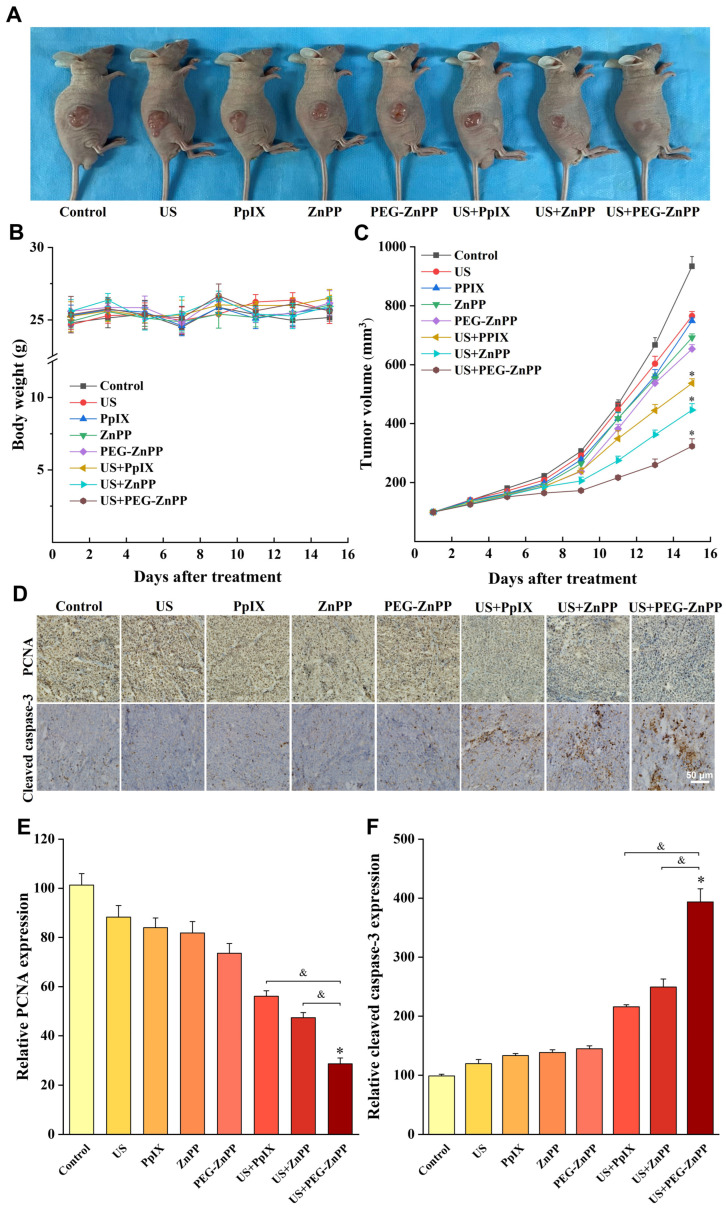
PEG-ZnPP-SDT exhibited significant antitumor effects in vivo. (**A**) Antitumor effects in ovarian cancer xenograft nude mouse model. (**B**) Plot of body weight versus days after different treatments. There were no significant side effects of SDT on nude mice in all groups. (**C**) Each curve represented the mean tumor volume of each group at different periods. The US + PEG-ZnPP group showed significant inhibitory effects on tumor growth compared with other groups. (**D**) Immunohistochemical detection of proliferation-associated PCNA and apoptosis-associated cleaved caspase-3 after different treatments. (**E**) PCNA protein expression was analyzed using IPP with OD values. The US + PEG-ZnPP group had significant inhibitory effects on the cell proliferation compared with other groups. (**F**) Cleaved caspase-3 protein expression was analyzed using IPP with OD values. The US + PEG-ZnPP group significantly induced cell apoptosis compared with other groups. * *p* < 0.05 vs. control group and ^&^ *p* < 0.05 between two groups. Data are the mean ± standard deviation of three independent experiments.

Finally, we evaluated the effects of PEG-ZnPP-SDT in vivo. As shown in Figure 7A,C, the average tumor volume in the US + PEG-ZnPP group was 323.4 ± 25.2 mm^3^ on the fifteenth day after SDT treatment, in which the tumor suppression effects were better than that in the US + PpIX and US + ZnPP groups (536.66 ± 15.3 mm^3^ and 446.0 ± 21.6 mm^3^). However, the drug alone or ultrasound alone groups showed no significant inhibitory effects on the tumor growth. In addition, the body weight curves of mice suggested that there were no significant side effects of SDT on nude mice (Figure 7B). Furthermore, the effects of PEG-ZnPP-SDT on the proliferation and apoptosis of ovarian cancer cells were evaluated. The PCNA expression (28.6 ± 2.4%) in the US + PEG-ZnPP group was significantly lower than that in the US + PpIX and US + ZnPP groups (56.1 ± 2.3% and 47.4 ± 2.1%), indicating that the US + PEG-ZnPP group had significant inhibitory effects on the proliferation of ovarian cancer (Figure 7D,E). The protein expression of the apoptosis marker cleaved caspase-3 (393.7 ± 22.2%) in the US + PEG-ZnPP group was higher than that in the US + PpIX and US + ZnPP groups (215.8 ± 3.4% and 249.3 ± 13.6%) (Figure 7D,F). These results indicated that PEG-ZnPP-SDT exhibited significant antitumor effects in ovarian cancer.

## 4. Discussion

Ovarian cancer is one of the most common malignant tumors in gynecology. In 2020, it is estimated that 313,959 new diagnoses and 207,252 deaths occurred from this neoplasm, which is the third most prevalent cancer and the second most lethal cancer in women in the world [1]. Because of its recurrence and drug resistance, the prognosis of ovarian cancer is poor, which seriously threatens the lives and health of women, leading to great challenges in the clinical treatment of ovarian cancer [19].

SDT is a promising physical therapy developed on the basis of photodynamic therapy (PDT). It generates reactive oxygen species (ROS) to kill tumor cells using ultrasound-activated sonosensitizers [3,20,21]. Ultrasound, sonosensitizer, and molecular oxygen are the three basic elements of SDT, which show synergistic antitumor effects. Especially, the great advantage of SDT is its deep tumor-penetrating ability compared with clinical PDT [12,22,23]. Furthermore, SDT can maintain the integrity of ovarian organs, preserve female reproductive ability, and improve women’s quality of life. Therefore, SDT will have good application prospects for ovarian cancer.

It is well known that sonosensitizer is the most critical factor that affects SDT outcomes. So far, various types of sonosensitizers have been used in preclinical research and are mainly based on porphyrins and their derivatives. The first-generation sonosensitizers, such as hematoporphyrin and photofrin, have complex components, a slower metabolism (up to 1 month), and severe phototoxic side effects, which hinder the clinical applications of SDT. Therefore, the second generation of sonosensitizers has been developed, which have the characteristics of a single component, a faster metabolism, and higher ROS production [21]. Sinoporphyrin sodium (DVDMS) had entered the phase III clinical trial because of the advantages of better water solubility, higher chemical purity, better targeting, and a shorter skin-sensitivity period [24]. DEG and DCPH-P-Na (I) exhibited not nearly as much phototoxicity compared with other sonosensitizers [25,26]. HMONs-MnPpIX-PEG could be used as a multifunctional sonosensitizer for magnetic resonance imaging (MRI)-SDT against cancer, besides having controllable biodegradation and high biocompatibility [16]. In a word, the emergence of these new sonosensitizers has significantly improved the therapeutic effects of SDT.

However, it cannot be ignored that ROS could be scavenged by the antioxidant system of tumor cells. ROS mainly include superoxide, hydrogen peroxide, singlet oxygen (^1^O_2_), and hydroxyl radicals [27]. Endogenous ROS are mainly byproducts of electron leakage from the mitochondrial respiratory chain. In addition, tumor cells can produce more endogenous ROS compared with normal cells, which may be caused by the activation of oncogenes, inactivation of tumor suppressor genes, aberrant cell metabolism, and mitochondrial dysfunction [28]. Therefore, tumor cells must require strong ROS-scavenging systems (such as HO-1, superoxide dismutase, catalase, peroxiredoxin, thioredoxin, glutaredoxin, and glutathione peroxidase) to eliminate ROS, in which the HO-1 antioxidant system plays an important role [28].

HO-1 is an inducible enzyme localized in the cellular microsomes and belongs to the heat-shock protein HSP32 family. It can be activated by multiple factors, such as oxidative stress, heat shock, ultraviolet light, heavy metals, and toxins [29]. It functions by degrading heme to generate CO, Fe^2+^, and bilirubin, which is the most critical initial and rate-limiting enzyme in the heme metabolism. All HO-1 products are important biological effector molecules; bilirubin is a very effective ROS scavenger. CO has significant anti-apoptotic and anti-inflammatory effects. Fe^2+^ can induce the production of ferritin and participate in the protection of cells [29]. In addition, HO-1 can promote tumor angiogenesis, cell proliferation, invasion, and metastasis, etc., and is highly expressed in various malignant tumors, including prostate cancer, kidney cancer, breast cancer, lung cancer, liver cancer, pancreatic cancer, melanoma, and especially in gynecological cancers (ovarian cancer, etc.) [30]. Studies have found that the expression of HO-1 increases during radiotherapy, chemotherapy, and even PDT treatment, and HO-1 plays a role in protecting cell survival [31,32]. Therefore, it is key to destroy the HO-1 antioxidant system in tumor cells in order to resist the elimination of ROS, thereby improving the efficacy of SDT.

Previous studies have found that zinc protoporphyrin (ZnPP) is a potent inhibitor of HO-1 activity and plays an antitumor role in various tumors [33,34,35]. ZnPP is a natural intermediate product in the heme metabolism, accounting for 90% of the total porphyrin in the human body. ZnPP has a stronger affinity with HO-1 and is more likely to destroy the antioxidant capacity of tumor cells by inhibiting the activity of HO-1 [36]. At the same time, studies have also shown that ZnPP can be used as a photosensitizer for the treatment of PDT [37]. Therefore, it has great potential to become a novel sonosensitizer. However, there have not been reports about ZnPP-mediated SDT (Znpp-SDT) for cancer treatment until now, and it is more difficult to elucidate the molecular mechanisms of Znpp-SDT in ovarian cancer.

Regrettably, ZnPP is prone to aggregation in aqueous solutions like other metalloporphyrins, which causes not only self-quenching, but also shorter blood circulation times and lower enrichment efficiency and is limited in practical application. In recent years, people have developed many methods of water-soluble modification, such as polyethylene glycol (PEG) [38], PLGA [39], albumin [40], of which the United States Food and Drug Administration (FDA) prefers PEG due to its higher biocompatibility and water solubility, lower immunogenicity, and lower toxicity [41]. In this study, we developed an improved water-soluble method to solve the low efficiency of polyethylene glycol (PEG)-conjugated metalloporphyrin and found that its high concentration (1 mM) maintained better water solubility (for a period of 30 days). In addition, we also found that due to the EPR effect (enhanced permeability and retention effect) in the tumor, the accumulation concentration of PEG-ZnPP in the mouse model of ovarian cancer xenograft was about 5–10 times higher than that in normal tissue (date not shown), which is consistent with other previous report [42].

It is believed that SDT-mediated apoptosis rarely induces a large-scale inflammatory response and has fewer side effects on normal cells compared with necrosis. Generally, there are two apoptotic pathways in cells: mitochondria-dependent apoptosis and death receptor-mediated apoptosis [43,44]. In this study, we found that PEG-ZnPP-SDT generated more than double the ROS yields compared with PpIX-SDT or ZnPP-SDT. The possible reasons were as follows: (1) PEG-ZnPP had better water solubility and stronger dispersibility, leading to higher ROS yields. (2) PEG-ZnPP could destroy the HO-1 antioxidant capacity in tumor cells, leading to inefficient elimination of ROS. In this study, it was confirmed that PEG-ZnPP-SDT promoted more significant apoptosis than that of PpIX-SDT and ZnPP-SDT in vivo and in vitro, and the tumor inhibitory effects of the PEG-ZnPP-SDT group were also more significant than those of the PpIX-SDT and ZnPP-SDT groups. In addition, it was reported that ROS could increase the permeability of the mitochondrial outer membrane and regulate the release of mitochondrial cytochrome c [45]. Subsequently, cytochrome c could bind to procaspase-9/Apaf-1, thereby activating the effector caspase-3 to initiate the mitochondrial–caspase apoptotic pathway. Hererin, it was confirmed that PEG-ZnPP-SDT could significantly increase ROS production, leading to the collapse of mitochondrial membrane potential in ovarian cancer SKOV3 cells, the leakage of cytochrome c from mitochondria to the cytoplasm, and finally the activation of the mitochondrial–caspase apoptotic pathway.

## 5. Conclusions

In this study, we developed an improved water-soluble method to solve the low efficiency of PEG-conjugated metalloporphyrin. Additionally, and more importantly, our results showed that PEG-ZnPP-SDT could effectively enhance ROS production by destroying the HO-1 antioxidant system in ovarian cancer. Increased ROS could cause mitochondrial membrane potential collapse, release cytochrome c from mitochondria to the cytoplasm, and trigger the mitochondrial–caspase apoptotic pathway. This study demonstrated for the first time that ZnPP, as a novel sonosensitizer, could be used to treat tumors in SDT, to elucidate in detail the molecular mechanism of PEG-ZnPP-SDT in the treatment of ovarian cancer, and to prove that PEG-ZnPP-SDT could improve antitumor effects by destroying the HO-1 antioxidant system. It provided a new therapeutic strategy for SDT to treat cancers, especially those with higher HO-1 expression.

## Data Availability

The data that support the findings of this study are available from the corresponding author upon reasonable request.
